# Measuring Behaviors and Identifying Indicators of Self-Regulation in Computer-Assisted Language Learning Courses

**DOI:** 10.1186/s41039-018-0087-7

**Published:** 2018-12-05

**Authors:** Huiyong Li, Brendan Flanagan, Shin’ichi Konomi, Hiroaki Ogata

**Affiliations:** 1 Graduate School of Informatics, Kyoto University, 36-1 Yoshida-Honmachi, Sakyo-ku, Kyoto, 606-8501 Japan; 2 Academic Center for Computing and Media Studies, Kyoto University, Kyoto, Japan; 3 Faculty of Arts and Science, Kyushu University, Fukuoka, Japan

**Keywords:** Self-regulated learning (SRL), Trace measures of SRL, Computer-assisted language learning, Learning analytics, Learning types

## Abstract

The aim of this research is to measure self-regulated behavior and identify significant behavioral indicators in computer-assisted language learning courses. The behavioral measures were based on log data from 2454 freshman university students from Art and Science departments for 1 year. These measures reflected the degree of self-regulation, including anti-procrastination, irregularity of study interval, and pacing. Clustering analysis was conducted to identify typical patterns of learning pace, and hierarchical regression analysis was performed to examine significant behavioral indicators in the online course. The results of learning pace clustering analysis revealed that the final course point average in different clusters increased with the number of completed quizzes, and students who had procrastination behavior were more likely to achieve lower final course points. Furthermore, the number of completed quizzes and study interval irregularity were strong predictors of course performance in the regression model. It clearly indicated the importance of self-regulation skill, in particular completion of assigned tasks and regular learning.

## Introduction

More than three decades ago, research into the self-regulation of academic learning and performance emerged to answer the question of how students become self-regulated learners. Initial attempts to measure self-regulated learning (SRL) using questionnaires and interviews were successful in demonstrating significant predictions of students’ academic outcomes (Pintrich et al. 1993; Zimmerman and Pons 1986; Schunk and Zimmerman 2012). Recently, research on SRL has evolved to develop online measures of self-regulatory processes regarding learning in authentic learning contexts. Unlike self-reported measures in traditional learning contexts, online measures focus on assessing self-regulated learning processes and are based on actual learning behaviors in authentic contexts. Innovative online measures of SRL offer detailed information concerning the interrelation of various processes in real time, such as computer traces (Winne et al. 2006).

In higher education, online learning has fewer restrictions and allows students to learn at any time and in any place. However, the lower constraints of this learning setting necessitate self-regulation by students and intrinsic motivation (Goda et al. 2015; Lynch and Dembo 2004; Rakes and Dunn 2010). Online learners are required to self-manage their learning processes and be responsible for initiating, planning, and conducting their studies. Previous research has shown that failure to study regularly leads to poor academic achievement, and procrastination and withdrawal have been proven to be persistent problems in online learning (Elvers et al. 2003; Michinov et al. 2011). Therefore, the way in which strategic support and self-regulation of online learners can influence learning should be investigated to keep students motivated, regulated, and participating in their courses.

Learning records provide new opportunities to monitor students’ learning process, as students’ learning behaviors in an online learning environment can be automatically recorded by online learning systems. According to previous studies utilizing students’ log data, frequency measures are the most typical parameters used to explain individual differences in online learning (Morris et al. 2005). However, several studies claimed that frequency counts of events were minimally relevant to engaged learning, and they are limited to suggesting instructional interventions and providing practical learning guidance (Hrastinski 2008; Kirkwood and Price 2014). To further understand the process of SRL, more elaborate time-based indicators from students’ log data should be defined.

In this context, this study aims to measure self-regulated behaviors in computer-assisted language learning (CALL) courses and identify typical learning patterns, such as procrastination and regular learning. Three novel time-based measures are proposed: (i) a measure of “anti-procrastination,” that is, whether a student completes the materials in advance at each course stage and how early the student completes learning materials; (ii) a measure of “irregularity of study interval,” which means a standard deviation of study intervals per student on a daily basis; and (iii) a measure of the “pacing” of access, that is, whether a student is keeping pace with the prescribed flow of materials as the course proceeds.

Moreover, this study also examines the relationship between behavioral indicators and learning outcomes, which would contribute to identifying effective self-regulated behaviors in an online learning environment.

Our research project aims to develop a learning support system for CALL courses to provide appropriate and customized feedback in a timely manner based on students’ actual learning behaviors. This study, which is as a part of the project, is positioned to measure behaviors and identify indicators of self-regulation and to determine the appropriate timing for such learning support.

## Related work

### SRL in computer-assisted environments

SRL is an active and constructive process through which learners can set goals and monitor and control their cognition, motivation, and behavior (Pintrich 2000). It is also characterized as a self-directive process, as self-beliefs enable learners to transform their academic abilities (Zimmerman 2008). Winne and Hadwin (1998) proposed that SRL included four phases: defining the task, setting goals and plans, enacting tactics, and adapting metacognition. Therefore, learners need to analyze the learning context and define tasks, set the appropriate learning goals and make plans, select the effective learning strategies to use, monitor the whole learning process, and evaluate their learning performance.

Previous studies indicated that SRL is a crucial skill for success in computer-assisted environments (Adeyinka and Mutula 2010). However, learners cannot always regulate themselves successfully because of reasons, such as lack of good strategy use, lack of metacognitive knowledge, failure to control metacognitive processes, or lack of experience in learning environments with multiple representations.

Thus, how to foster SRL ability has become a central issue in the field of education research and practice. In order to support learner’s acquisition of self-regulation skills in CALL courses, instruments that capture students’ self-regulation are critical. Most studies on self-regulated learning have used self-reported instruments, which not only are intrusive but also are limited to capturing self-regulated behaviors in computer-assisted environments. However, as mentioned earlier, this issue can be resolved through the use of online trace data, and technologically mediated learning environments enable the collection of a comprehensive set of student learning behaviors that occur (Pardo 2014).

### Learning analytics for SRL

As Winne and Baker (2013) noted, “Self-regulated learning is a behavioral expression of metacognitively guided motivation.” Consequently, every trace records a motivated choice about how to learn. Analyzing trace data could better understand and discover meaningful behavioral patterns about rate of progress, effort spent, or time management.

Numerous studies have reported the benefits of utilizing learning analytics (LA) in terms of examining online course performance (Johnson 2005; Morris et al. 2005; DietzUhler and Hurn 2013). These results imply that active participation is essential to successful online learning. Furthermore, a few studies have focused on the quality of learning rather than the number of online participation (Asarta and Schmidt 2013; Cheng and Chau 2016). Asarta and Schmidt were particularly interested in the timing dimension of access to 36 online lesson materials. They examined the effect of timing, volume, intensity, and consistency of access on achievement. They clarified that keeping pace with the class schedule, studying the materials in advance of an exam without cramming, and accessing course materials regularly are vital factors for achievement. These findings support the notion that various characteristics of learning behaviors rather than simply the frequency of access should be taken into account.

Despite a growing body of research that examines interpreting online engagement to support the learning process in online learning environments, little is known on how to measure self-regulated learning and to examine the effects on course success. Yet, interpreting and evaluating qualities of actions, strategies, goals, and more broadly regulation is a much more challenging task (Roll et al. 2014). Developing indicators of self-regulated learning is the first step to addressing this challenge. The extraction and aggregation of meaningful indicators should support understanding of students’ learning statuses and providing actionable feedback.

## Methods

In this section, we examine the trace data and focus on self-regulated behavioral indicators in CALL courses. There are two main research questions in this study:

Research question 1. What learning behavioral patterns exist in the trace data in CALL courses?

Research question 2. Which behavioral factors significantly predict the final course point?

### Setting and participants

Fifty mandatory CALL courses at a national university in Japan were employed in this research. The courses were provided to freshman students for self-regulated learning from the spring semester to the fall semester. Table 1 shows the course schedule for 1 year. To increase students’ motivation, four sub-deadlines were set in each semester. Students were required to complete the assigned materials from the first stage to the third stage, with the fourth stage as an optional one in each semester.

**Table 1:** Course schedule for 1 year

Semester	Stage	Deadline	Learning materials assigned		
			Reading	Listening	Grammar
Spring	1	Week 5	Reading1	Listening1	Grammar1
	2	Week 10	Reading2	Listening2	Grammar2
	3	Week 15	Reading3	Listening3	Grammar3
	4 (optional)	Week 21	Reading4	Listening4	Grammar4
Fall	5	Week 30	Reading5	Listening5	Grammar5
	6	Week 36	Reading6	Listening6	Grammar6
	7	Week 42	Reading7	Listening7	Grammar7
	8 (optional)	Week 47	Reading8	Listening8	Grammar8

The e-learning materials of the CALL course contained grammar, listening, and reading sections. A total of 973 quiz units were included, with 493 quiz units in the spring semester and 480 quiz units in the fall semester, respectively. The difficulty of e-learning materials increased stage by stage. Table 2 indicates the details of the e-learning materials.

**Table 2:** Categories and unit numbers of learning materials

Section	Part	Category	Unit #
Reading	1	Reading comprehension	6
	2	Reading comprehension	7
	3	Reading comprehension	6
	4	Reading comprehension	6
	5	Reading comprehension	6
	6	Reading comprehension	7
	7	Reading comprehension	6
	8	Reading comprehension	6
Listening	1	Short conversation	15
	2	Long conversation	14
	3	Long announcement	15
	4	Formal conversation	22
	5	Short conversation	15
	6	Long conversation	14
	7	Long announcement	14
	8	Formal conversation	21
Grammar	1	Grammar and word usage	95
	2	Grammar and word usage	89
	3	Grammar and word usage	98
	4	Grammar and word usage	120
	5	Grammar and word usage	84
	6	Grammar and word usage	81
	7	Grammar and word usage	106
	8	Grammar and word usage	120
		Total	973

A total of 2631 freshman students participated in this study. Students were from all departments of the Art and Science faculties. Eighty-four students (3.19%) dropped out and did not access the learning materials at all during the whole year. Additionally, 93 students (3.53%) who scored 520 or more in the semester-initial TOEFL-ITP exam applied for exemption from the CALL course. Thus, the total data used in this study were from the remaining 2454 students (93.27%).

### Data collection and measures

The freshman students conducted the CALL course on a language learning management system named WebOCM, and the system had a function for tracing students’ events. As students practiced quizzes online, the learning events were recorded in the server logs concurrently. Therefore, the trace data was retrieved from the server of the CALL course. There were three types of trace logs including access to learning materials (access logs), completed quiz items (completion logs), and quiz answers (answer logs). A total of 14,329,172 learning logs were restored for 1 year with 3,344,215 access logs, 2,199,340 completion logs, and 8,785,617 answer logs, respectively.

**Fig. 1:**
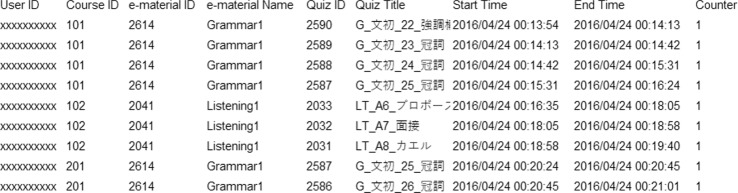
An example of access logs

An example of the raw data contained in access logs is shown in Fig. 1. The access logs presented information about the frequency and duration of actual learning behaviors, with columns such as user ID, quiz ID, start time, and end time. Besides these columns, a complete flag was included in the completion logs, and each answer for quizzes was stored in the answer logs.

The behavioral measures from the raw data used in this study were as follows:

- Number of completed quizzes
- Total access time
- Reviewing time
- Score of completed quizzes
- Anti-procrastination
- Irregularity of study interval
- Pacing

All learning variables in this study are summarized in Table 3. Variables 1 to 7 are behavioral measures derived from the raw data, and variables 8 and 9 are used for course achievement.

**Table 3:** Summary of learning variables

Variables	Description
1. Number of completed quizzes	The number of quizzes a student has completed
2. Total access time (h)	The total hours spent on accessing learning materials
3. Reviewing time	The total hours spent on reviewing learning materials
4. Score of completed quizzes	An average score of all quizzes which a student has completed
5. Anti-procrastination	A degree of how early a student completes quizzes
6. Irregularity of study interval (days)	A standard deviation of study intervals
7. Pacing	A count of the number of quizzes which are completed as assigned
8. Mid course point	The exam point in the spring semester
9. Final course point	The exam point in the fall semester

Of particular interest in this study is the measuring of self-regulation patterns from the trace data such as procrastination and regular learning. Thus, three measures were specifically created to identify self-regulation patterns.

**Fig. 2:**
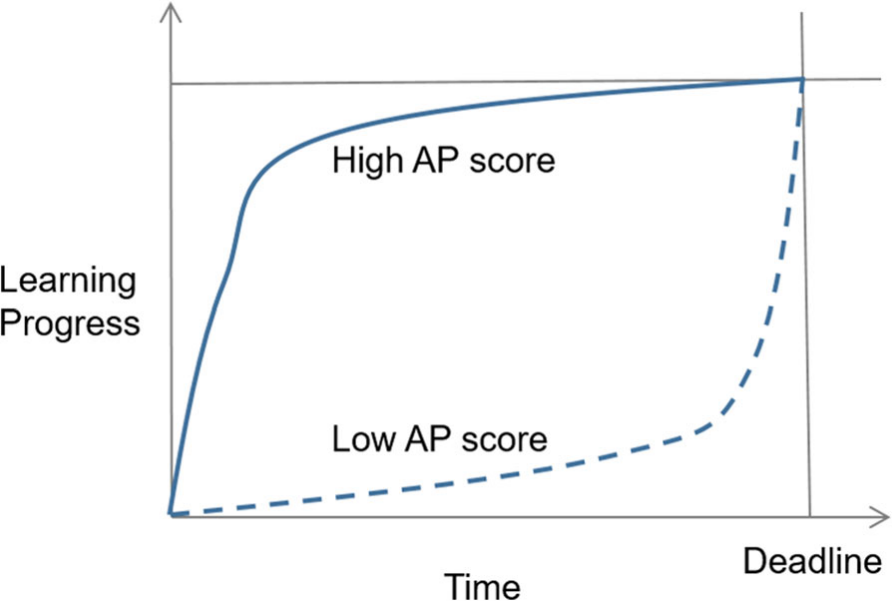
An example of high and low anti-procrastination (AP) scores

The first measure is “anti-procrastination.” It is calculated by comparing the total available days and the lead days when each quiz unit was completed, as shown in Eq. 1.

1 $$ AP=\sum\limits_{i=1}^{N} \frac{1}{N} * \frac{D-D_{a_{i}}}{D}  $$

where *N* is the number of completed quizzes, *a*_*i*_ is one quiz unit, ${D_{a_{i}}}$ is the days between the completed day of the quiz unit and the first day of the stage when the quiz unit is completed, and *D* is the total days of the stage. For each quiz unit, a score ranging from 0 to 1 is decided by comparing the completed day with the first day of the related stage. As shown in Fig. 2, the student who completed all quiz units just at the first day of each stage would receive the highest possible value of 1; however, one who completed all quiz units just before the deadline of each stage would receive the lowest value of 0. Therefore, the anti-procrastination measure was used to determine whether the students completed the quiz units in advance and how early the students completed the quiz units.

The second measure is the irregularity of study interval. It manifests how study intervals are dispersed. First, a set of daily activities of accessing learning materials were extracted per student. The study intervals in daily activities were then calculated. Finally, the standard deviation of study intervals per student was computed. A student who regularly learns would get a low score of the irregularity of study interval measure. This measure was used to determine the degree of continuous learning.

The third measure is “pacing.” It refers to a count of the number of quiz units that were completed as assigned. As noted earlier, a course schedule informed students of the online materials that should be completed before the four given sub-deadlines. Ideally, students should complete quiz units stage by stage rather than cramming with all quiz units within several days. Thus, a value of 1 would be recorded to the pacing measure if the student completed one quiz unit within the scheduled stage. A high pacing measure would indicate that the student was keeping the learning pace as the assignment schedule. Since the number of quiz units was 973, the cumulative measure ranged from 0 to 973.

Moreover, the total access time is a broad measure of online activities and was calculated by summing the total time spent on accessing learning materials. The reviewing time is the cumulative time spent on reviewing learning materials. The number of completed quizzes is referred to the degree of course completeness. The score of completed quizzes is an average score of all quizzes which the student completed.

Finally, two exam points were used to evaluate the effects of learning pace patterns on student performance. The examinations were administered through an offline campus at the end of the spring and fall semester, respectively. They were graded in the form of five letter grades: A, B, C, D, and F. This grading scale is commonly used, where topical grades where A ranks the highest and F, short for failed, is the lowest. For the sake of easy computation, the grades of A, B, C, D, and F were digitized as 4, 3, 2, 1, and 0, respectively. The results of the first exam conducted in the spring semester are referred to as the mid course point, while the results of the second exam conducted in the fall semester are treated as the final course point.

### Data analysis

To investigate the research questions, three phases of analysis were conducted.

First, descriptive statistics were performed for all behavioral measures, including “anti-procrastination,” the irregularity of study interval, and “pacing.”

Second, clustering analysis was applied to find answers to the first research question. The differences of learning pace would be examined based on “anti-procrastination” and the number of completed quizzes. The *k*-means algorithm was used to extract clusters from these two measures.

Finally, hierarchical regression analysis was chosen to identify significant behavioral measures related to course achievement. In the process of hierarchical regression analysis, a stepwise method was conducted. A significance level of.05 was used to test the hypothesis.

## Results

In this section, we first discuss the results of descriptive statistics for all behavioral measures. Then, the result of clustering analysis is described. Finally, the model of hierarchical regression analysis for course achievement will be proposed.

### Descriptive statistics

Table 4 contains descriptive statistics for all behavioral variables. The mid course point (*M* = 3.3, SD = 1.0) and final course point (*M* = 3.2, SD = 1.1) had high mean values and indicates that the majority of students completed the course with high points. Additionally, the reviewing time (*M* = 2.8, SD = 4.3) and anti-procrastination (*M* = 0.27, SD = 0.14) varied widely. The distribution of the score of completed quizzes (Skewness = − 0.28) was close to a normal distribution, whereas the distribution of the irregularity of study intervals (Skewness = 2.01) was skewed to positive. The results revealed that the majority of students completed the course with wide differences in learning pace as well as time management.

**Table 4:** Descriptive statistics of the behavioral variables (*n* = 2454)

Variables	Mean	SD	Min.–max.
1. Number of completed quizzes	800.4	160.3	2–973
2. Total access time (h)	21.2	11.6	0.01–109.48
3. Reviewing time	2.8	4.3	0–60
4. Score of completed quizzes	65.6	12.1	0–98
5. Anti-procrastination	0.27	0.14	0.03–0.84
6. Irregularity of study interval (days)	16.6	8.3	0–90
7. Pacing	742.5	166.7	2–973
8. Mid course point	3.3	1.0	0–4
9. Final course point	3.2	1.1	0–4

### Results of clustering analysis

Two measures were used in the cluster analysis: anti-procrastination and number of completed quizzes.

In order to determine the optimal number of clusters for the *k*-means algorithm, two main evaluation methods were computed: the elbow method and the silhouette method. According to the resulting evaluation, 7 was chosen as the optimum number of clusters.

The average of the clusters are given in Table 5. Cluster 1, cluster 2, and cluster 4 accounted for nearly half of the students (*n* = 1163, 47%), and they completed the course tasks in the last few days before each deadline. The behavior is known as procrastination, which means the delay of initiation or of completion of important tasks. The final course point average in three clusters increased with the number of completed quizzes. Besides, the students who reached the equal number of completed quizzes acted at different learning paces.

**Table 5:** Average of the clusters for learning pace

	*n*	Number of completed quizzes	Anti-procrastination	Final course point
Cluster 1	526	674	.16	2.79
Cluster 2	558	870	.16	3.42
Cluster 3	282	924	.52	3.80
Cluster 4	79	360	.15	1.24
Cluster 5	529	754	.35	3.11
Cluster 6	38	298	.50	2.05
Cluster 7	442	961	.30	3.85

**Fig. 3:**
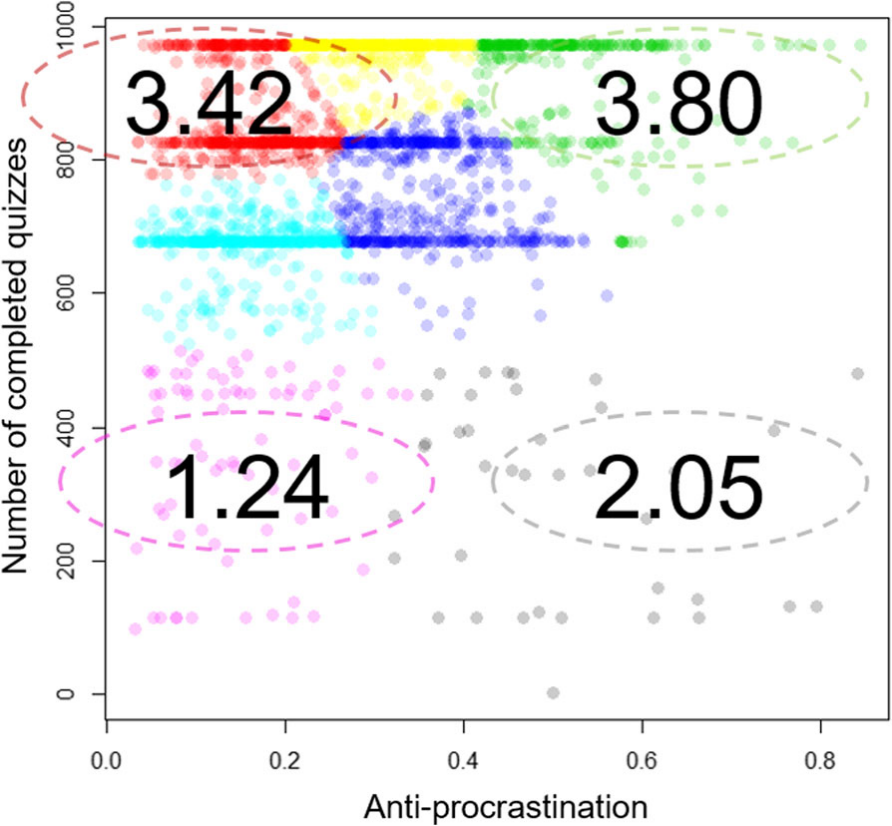
Cluster distributions for learning pace and the final course point average of four clusters

Out of seven original clusters, four typical groups for learning pace were identified: Early Completers, Late Completers, Early Dropouts, and Late Dropouts. The cluster distributions and the final course point average of four clusters are shown in Fig. 3.

*Cluster green: Early Completers*

This cluster includes students who started to access online materials at the early days of each stage and finally completed the required learning materials. Early Completers accounted for 11% of students in the course. They received an average of 3.80 final course points.

*Cluster red: Late Completers*

This cluster contains students who rushed to access online materials just before the last days of each stage and finally completed the required online materials. Late Completers made up the largest cluster, accounting for 23% of students in the course. They received an average of 3.42 final course points, which was 0.38 lower than Early Completers (*p* <.001).

*Cluster black: Early Dropouts*

These students started to access online materials at the early days of each stage but then dropped out of the course. Early Dropouts made up 2% of students in the course with an average of 2.05 final course points.

*Cluster pink: Late Dropouts*

These students rushed to access online materials just before the last days of each stage but failed to complete the required online materials. Late Dropouts made up 3% of students in the course with the lowest average of 1.24 final course points.

### Results of hierarchical regression analysis

Hierarchical regression analysis was conducted to predict the final course point. The following variables were analyzed in the prediction: the number of completed quizzes, total access time, reviewing time, the score of completed quizzes, anti-procrastination, irregularity of study interval, and pacing. Furthermore, the mid course point was also selected as a predictor.

Results are shown in Table 6. The number of completed quizzes (*B* =.002, *p* <.001), the mid course point (*B* =.265, *p* <.001), irregularity of study interval (*B* = − .022, *p*<.001), the score of completed quizzes (*B*=.010, *p*<.001), total access time (*B*=.010, *p*<.001), and pacing (*B*=.001, *p*<.001) were significant. The regression model explained 40.5% of the variance in the final course point (*R*^2^=.405, *F* (6, 203) = 274, *p*<.001). Note that the reviewing time measure was not significant since it was removed from the modeling process. The *R*^2^ value was slightly greater than 40% and is not so high to conduct precise course achievement prediction. However, this is an acceptable value when taking into account the fact that there is a large variation of personal behaviors.

**Table 6:** Hierarchical regression analysis results on the final course point

Model	Predictors	Final course point			
		B	SE	*β*	*R* ^2^
M6	Number of completed quizzes	.002	.000	.230^∗∗∗^	.405
	Mid course point	.265	.021	.231^∗∗∗^	
	Irregularity of study interval	− .022	.003	− .158^∗∗∗^	
	Score of completed quizzes	.010	.002	.104^∗∗∗^	
	Total access time	.010	.002	.104^∗∗∗^	
	Pacing	.001	.000	.116^∗∗∗^	

*** *p*<.001

A beta coefficient compares the strength of the effect of one behavioral variable to the final course point. The higher the absolute value of the beta coefficient, the stronger the effect. The result revealed that the number of completed quizzes (*β*=.230), the mid course point (*β*=.231), and irregularity of study interval (*β*=− .158) were the most important predictor variables.

## Discussion

### Implications

The present findings have implications for self-regulated learning in the context of CALL and similar online learning environments.

First, this study contributes to the identification of unconventional but more relevant self-regulated learning measures from the trace data and studies their effectiveness. The “anti-procrastination” variable is considered as an elaborate measure regarding learning pace. It is based on the timing of when a quiz is completed and then transforms the behaviors into a number. This variable could also be considerable in other online courses as a quiz could be extended to a task and a learning stage could be set to specific days. Future work could use this variable to easily identify procrastination so that the instructors would further understand their students’ learning status.

Second, the measures of irregularity of study interval and pacing proved to be positive influence upon student performance. These findings support those of previous research, which has emphasized the quality of learning behaviors rather than the quantity of learning (Asarta and Schmidt 2013; You 2015; Cheng and Chau 2016). The results are consistent with accounts from prior research in online courses. Successful students actively participate in their learning in terms of regularly accessing course notices, carefully studying and reviewing the course content, completing the assignments in a timely manner, and self-evaluating their learning. By contrast, unsuccessful learners are characterized by their failures in estimating the amount of time and effort required to complete tasks and their lack of time-management and life-coping skills (Yukselturk and Bulut 2007).

Furthermore, these findings could be a foundation of further support for individuals during the whole self-regulated learning process. At the early stage of learning, these measures could be used to categorize learners and identify at-risk students based on their online action. For example, the students who are categorized as procrastinators could be periodically reminded to access the online materials at the remaining stages. At the end of learning, these measures are helpful to evaluate self-regulated learning behaviors for learners as well as for instructors. For example, a score of self-regulation could be sent to facilitate students’ self-reflection by integrating learning pacing, consistency, and completeness.

### Limitations

Although the present study demonstrates the benefits of identifying significant measures from trace data to facilitate self-regulated online learning, several limitations should be noted.

First, the e-learning materials used in this research were developed for those learning English as a foreign language. Therefore, subject matter and cultural context might have affected the behavioral patterns of the study.

Second, the data of actual learning was collected from a mandatory course. The nature of courses, mandatory or elective, might affect learners’ motivation and decision-making.

Third, the learning patterns should also be tested in relation to other variables, such as prior knowledge of English, motivation, and online learning experience. These types of background information have been suggested to increase the predictability of students’ performances.

Finally, the relationships between learning behavioral measures and course achievement that were obtained in this study were based on correlations and do not necessarily indicate causation. As such, these results should be cautiously interpreted.

## Conclusion

This study provided a quantitative account of self-regulated learning in CALL courses and advances the understanding of what learning behavioral patterns exist and which behavioral factors in the trace data can significantly predict the final course point. The results were based on log data from 2454 freshman university students over the period of 1 year. Because self-regulated learning is essential to online learning, measures that reflect the degree of self-regulation were specifically created, including anti-procrastination, irregularity of study interval, and pacing.

The results of clustering analysis revealed that students who took late action were more likely to achieved lower final course points. For learning pace, nearly half (47%) of students were procrastinators. In general, procrastination may lead to dropouts and can have negative effects on academic performance.

The regression model based on six variables explained 40.5% of the variance in the final course point. The number of completed quizzes and irregularity of study interval were strong predictors of course achievement. This clearly indicates the importance of self-regulation skill, in particular completion of assigned tasks and regular learning.

Based on these results, it is feasible to provide feedback and support for online learning to encourage students to form a learning habit. How to facilitate self-monitoring for students and how to promote self-regulated skills should also be considered in future work.
